# Supplementary material for: Single-cell Analysis of Intracellular Transport and Expression of Cell Surface Proteins
Source: bioRxiv. 2025 Jun 12:2025.06.09.658704. Preprint. [Version 1] doi: 10.1101/2025.06.09.658704 (PMC12259062; doi:10.1101/2025.06.09.658704)

bioRxiv preprint doi: https://doi.org/10.1101/2025.06.09.658704; this version posted June 12, 2025. The copyright holder for this preprint (which was not certified by peer review) is the author/funder, who has granted bioRxiv a license to display the preprint in perpetuity. It is made available under aCC-BY 4.0 International license.

Supplementary Methods:

GO Term Selection for ICT Gene Identification

To identify Gene Ontology (GO) terms associated with intracellular protein traffic, we used a targeted keyword strategy across the three GO domains: Biological Process (BP), Molecular Function (MF), and Cellular Component (CC). For BP, we selected GO terms containing keywords such as “intracellular transport,” “intracellular protein transport,” and “vesicle-mediated transport” to capture processes involved in intracellular trafficking and protein localization. For MF, we included terms matching “protein carrier activity,” “folding chaperone,” and “chaperone binding,” representing molecular functions linked to protein transport and stabilization. For CC, we identified terms associated with key trafficking organelles and compartments using keywords including “vacuole,” “autophago,” “endoplasmic,” “lysosome,” “Golgi,” “endosome,” “vesicle,” “vesicular,” “plasma membrane,” “intermediate compartment,” and “microtubule.” We then expanded each selected term to include all hierarchical descendants using the GO term ontology graph.

To ensure specificity for intracellular transport, we excluded GO terms unrelated to trafficking but potentially captured by overlapping keywords. These included terms related to phagocytosis, immune and neurosecretory granules, neurotransmitter exocytosis, and secretory processes such as histamine or protein secretion. Genes were retained only if they were annotated to both relevant GO terms in the BP or MF domains and localized to ICT-relevant compartments in the CC domain. This curated set of GO terms was used to define the ICT gene list for downstream analyses.

bioRxiv preprint doi: https://doi.org/10.1101/2025.06.09.658704; this version posted June 12, 2025. The copyright holder for this preprint (which was not certified by peer review) is the author/funder, who has granted bioRxiv a license to display the preprint in perpetuity. It is made available under aCC-BY 4.0 International license.

Supplementary Figures:

Figure S1. Comparison of dendrograms derived from η_2 coefficients with those derived from pairwise correlations of mRNA expression for the coding genes in (A) Severe and (B) Mild COVID-19.

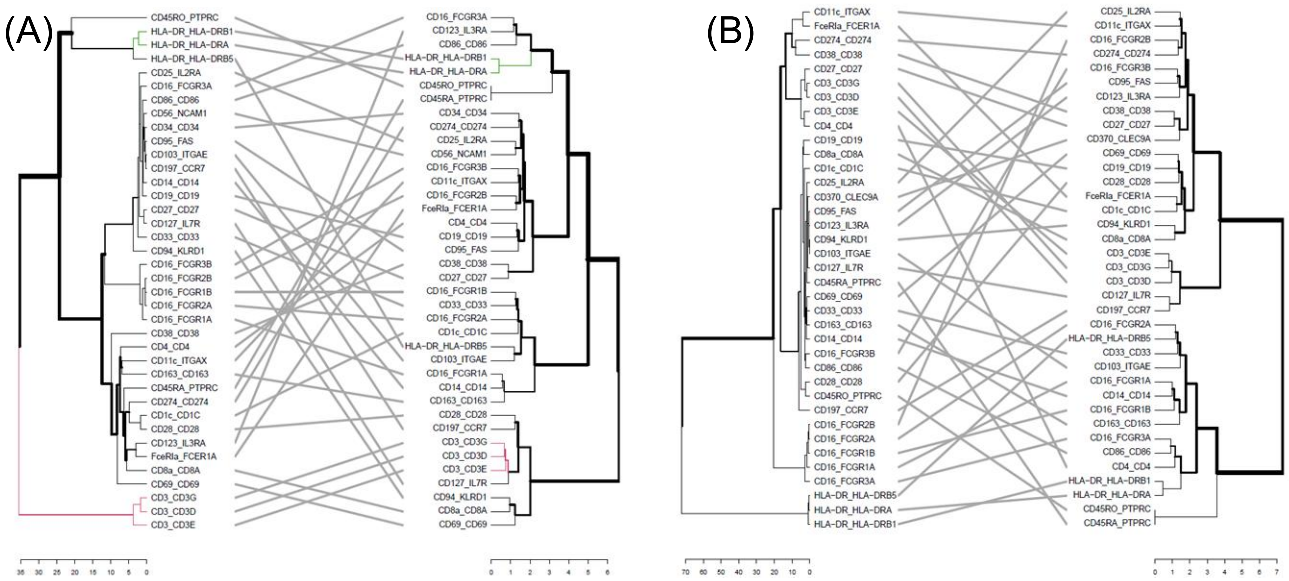

Figure S2. Comparison of the number of daICTs identified by Module II and Module III for each DEP in the severe vs. healthy comparison.

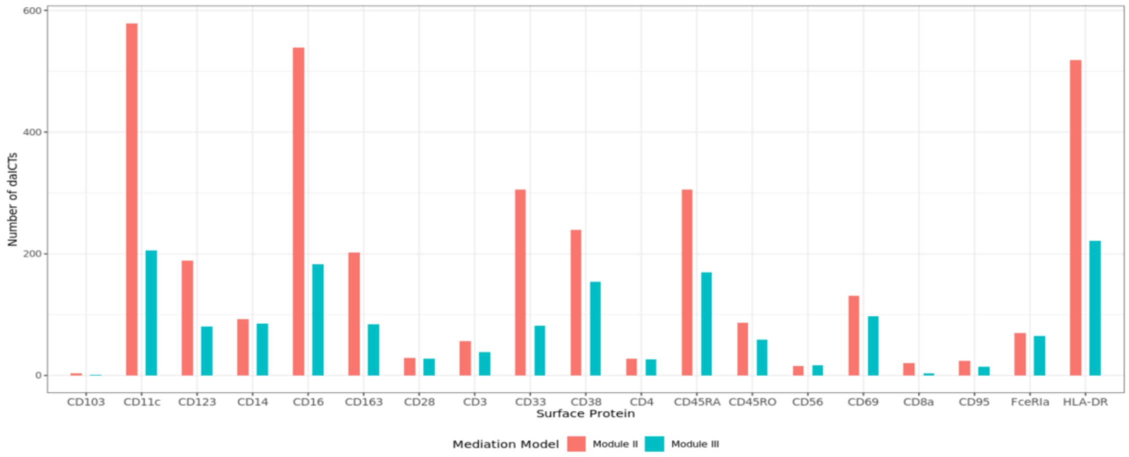

Supplement: 1 [file NIHPP2025.06.09.658704V1-supplement-1.pdf]
